# Synthesis of zeolite membranes on calcium silicate support and their bioactive response

**DOI:** 10.1007/s40204-018-0085-2

**Published:** 2018-02-10

**Authors:** A. Medina-Ramírez, A. A. Flores-Díaz, B. Ruiz Camacho, G. García-Ruiz

**Affiliations:** 1 Division de Ciencias Naturales y Exactas, Chemical Engineering Department, Universidad de Guanajuato, Campus Guanajuato. Noria Alta s/n, 36050 Guanajuato, Guanajuato Mexico; 2 Nanotechnology Engineering, Universidad de La Ciénega del Estado de Michoacan de Ocampo, Av. Universidad #3000 Lomas de la Universidad, 56020 Sahuayo, Michoacan Mexico

**Keywords:** Membrane, Zeolite, Bioactivity, Calcium silicate, Second growth, Cationic polymer

## Abstract

The synthesis of calcium silicate supported zeolite membrane was carried out by second growth method. The chemical nature of the functionalizing agent on the formation of homogenous zeolite membrane was evaluated. One monomer and two cationic polymers were used: 3-aminopropyltriethoxysilane (APS), polyethylenimine (PEI) and polydiallyldimethylammonium chloride (PDDA). The support was subjected to chemical functionalization and then it was rubbed with zeolite crystals. The W zeolite was used as zeolite seed in two different Si/Al ratios. The functionalized and rubbed supports were submitted to hydrothermal treatment at 150 °C for 48 h. The bioactivity of the homogeneous zeolite membranes was evaluated by the biomimetic method through the membranes soaking in a simulated body fluid (SBF) at 37 °C for 21 days. Two immersion methods were evaluated. The products were characterized by XRD and SEM techniques. The results indicated that the supported functionalization with PDDA and the Si/Al ratio (higher than 1.8) of zeolite enhanced the interaction between the support and the zeolite precursor enhancing the formation of homogeneous zeolite membrane on the surface. The presence of the functional groups of PDDA on the membrane was detected by FTIR. After immersion in SBF, the zeolite membrane was stable and led to the formation of Ca–P layer on its surface. The re-immersion method led to the formation of richer Ca/P layer (1.36). These findings allowed generating a zeolite membrane with combined properties of calcium silicate and the controllable porosity of zeolitic material making it potentially useful for bone regeneration and drug releasing.

## Introduction

The development of novel biomaterials with specific properties has intensified in the last years. Particularly the biocompatibility, stability and bioactive response are desirable properties in this kind of materials. Calcium silicates have been considered as candidate for bone regeneration and drugs carriers due to their bioactivity and degradability (Islam et al. 2015). Additionally the synergic effect of calcium oxide and silica structures can secure high bioactivity and clinical safety (Saravanapavan et al. 2003). Recently, the zeolites, the highly crystalline aluminosilicates, have attracted the attention for biomedical applications beyond their traditional applications like adsorbents, catalyst and ionic exchangers. Particularly, the membranes and nanoparticles of zeolites are being explored for biomedical applications. The zeolite-based nanostructured materials offer the potential to organize matter and to manipulate molecules with high spatial precision at nanometer level (Byrappa and Yoshimura 2001). The interest in the zeolites in the biomedical area is due to (a) these materials have known biological properties alone with long-term chemical and biological stability; (b) they reversibly bind small molecules such as oxygen and nitric oxide; (c) they possess size and shape selectivity; (d) they offer the possibility of metalloenzyme mimicry and (e) they have immunomodulatory activity (Pavelic and Hadzija 2003).

Diverse investigations have been focused on the study of zeolites and zeolite composite for biomedical applications, namely: polymeric nanofibers–ZSM5 zeolite composite membranes being evaluated in the creatinine uptake (Lu et al. 2016), hybrid membranes (polymer–zeolite) employed as a platform of ibuprofen release (Salazar et al. 2015), zeolite membranes evaluated as scaffolds (Tavolaro et al. 2016a) and as a control of the neoplastic activity (Tavolaro et al. 2016b) and zeolite immobilized on nanofiber as antimicrobial (Rieger et al. 2016), zeolitic imidazole frameworks for ATP imaging in live cells (Deng et al. 2017), and zeolite composite membranes as vector drug delivering for famotidine evaluating mordenite (MOR) and MFI zeolites on stainless steel supports (Tavolaro et al. 2013). A titanium alloy coated with MFI zeolite was found that the zeolite coating enhanced the osteointegration and osteogenenesis (Li et al. 2015). Hydroxyapatite–zeolite composite showed a good bioactivity and in vitro cell compatibility (Iqbal et al. 2014).

During the synthesis of zeolite membranes, factors such as the chemical nature of the substrate, the synthesis method and zeolite type were used to determine the properties of the membrane. Specifically the interaction between support and zeolite precursor is crucial for the formation of homogeneous membrane. Diverse studies have been focused on the preparation of the surface support through the incorporation of zeolite seeds (Yoo et al. 2010; Au et al. 2001) and the surface functionalization (Huang et al. 2010, 2012). According to these research works, the functionalization with cationic polymers promotes the covalent binding between the support and zeolite, enhancing the formation of zeolite membrane. Nevertheless, the success of this approach depends on the chemical nature of polyelectrolyte as well as the support. The chemical nature of the support influences zeolite nucleation, crystal growth and film adhesion (Chau et al. 2000). The synthesis of the Lynde type A zeolite membrane has been reported on alumina supports using polydiallyldimethylammonium chloride (PDDA) as covalent binding agent (Aguado et al. 2011), and also on α-alumina precoated with polyethyleneimine (Dey et al. 2013).

To the best knowledge of the authors, there are no reports about zeolite membrane growth on calcium silicate supports. For these reasons in the present work, we evaluated the feasibility of growth of zeolite membrane by second growth on this substrate. The effect of the support functionalization with three cationic polymers followed by seeding process on zeolite membrane formation was evaluated. The bioactivity of the zeolite membrane was evaluated by biomimetic method using a simulated body fluid. We proposed to take advantage of the properties of calcium silicate and W zeolite in order to generate a zeolite membrane with bioactive properties that can be useful for bone regeneration.

## Experimental

### Materials

Calcium silicate (87% SiO_2_) was used as support. Colloidal silica (Ludox HS-40) and sodium aluminate were used as precursors for the zeolite synthesis. Potassium hydroxide (90%) was used as an alkaline agent. One monomer and two cationic polymers were used as functionalization agents for the surface support being 3-aminopropyltriethoxysilane (APS, 98%, *M*w 221.37 g mol^−1^), polyethylenimine (branched PEI, 50 wt% in water, average *M*_w_ ~ 1300) and polydiallyldimethylammonium chloride (PDDA, 20 wt% in H_2_O). All polyelectrolytes were used without further purification. All reagents were purchased from Sigma Aldrich™. For SBF solution NaCl (90%), NaHCO_3_ (98%) KCl (92%), K_2_HPO_4_·3H_2_O, (89%), MgCl_2_·6H_2_O, (90%), HCl (37%), CaCl_2_ (89%), Na_2_SO_4_ (87%) and (CH_2_OH)_3_ CNH_2_ (91%) were used. Deionized water was used as solvent and reaction media.

### Formation of zeolitic membranes

#### Preparation of W zeolite seeds

Two syntheses were performed with two different contents of sodium aluminate. Both were carried out according to the procedure reported by Strohmaier (2001). For the WA zeolite, a batch of molar composition 0.0574 K_2_O: 0.0701 SiO_2_: 0.0177 Al_2_O_3_: 1.0384 H_2_O was prepared and for the WB a gel of molar composition 0.0574 K_2_O: 0.0701 SiO_2_: 0.0275 Al_2_O_3_: 1.0384 H_2_O was prepared. Each of them was submitted to hydrothermal treatment in a Teflon-lined stainless steel autoclave of 43 mL of capacity. The crystallization was performed at 150 °C for 48 h. The products were recovered, filtered and washed with deionized water. Subsequently, the crystallization products were dried at 100 °C for 12 h.

#### Preparation of the substrates

A quantity of the calcium silicate was pressed in an ICL EZ-Press™ to obtain disks of 1 cm of diameter. The disks were submitted to heat treatment at 500 °C for 2 h.

#### Functionalization of the substrates

Solutions at 2% v/v in water were prepared for PDDA and PEI, while a solution of 2% v/v in ethanol was prepared for APS and they were stored at 4 °C. Then, the substrates were added to 10 mL of the polymeric solution and sonicated for 10 min. Afterwards, the disks were recovered and rubbed with 0.1 g of the W zeolite seeds to incorporate the zeolite crystals on the substrate surface. The rubbed substrates were gently washed with water or ethanol and then they were dried at 60 °C for 24 h.

#### Membranes growing process

Gels of batch composition corresponding to WA and WB zeolites seeds were prepared and they were transferred to Teflon-lined stainless steel autoclave. The functionalized and rubbed disks were collocated in a Teflon support home-made in the middle of autoclave. The supports were submitted to hydrothermal treatment at 150 °C for 48 h. Afterwards, the products were recovered, gently washed with deionized water and dried at 60 °C for 4 h. The summary of the experimental conditions for the synthesis of zeolite membranes is given in Table 1.

**Table 1:** Experimental conditions of the zeolite membrane synthesis on calcium silicate support

Exp	Cationic polymer	Seed zeolite	Batch composition	Membrane growing
ZMP1-WA	APS	WA	0.0574 K_2_O: 0.0701 SiO_2_: 0.0177 Al_2_O_3_: 1.0384 H_2_O	None
ZMP2-WA	PEI	WA	0.0574 K_2_O: 0.0701 SiO_2_: 0.0177 Al_2_O_3_: 1.0384 H_2_O	None
ZMP3-WA	PDDA	WA	0.0574 K_2_O: 0.0701 SiO_2_: 0.0177 Al_2_O_3_: 1.0384 H_2_O	Scarcely
ZMP1-WB	APS	WB	0.0574 K_2_O: 0.0701 SiO_2_: 0.0275 Al_2_O_3_: 1.0384 H_2_O	Scarcely
ZMP2-WB	PEI	WB	0.0574 K_2_O: 0.0701 SiO_2_: 0.0275 Al_2_O_3_: 1.0384 H_2_O	None
ZMP3-WB	PDDA	WB	0.0574 K_2_O: 0.0701 SiO_2_: 0.0275 Al_2_O_3_: 1.0384 H_2_O	Homogeneous

### Bioactivity evaluation

To evaluate the bioactivity of the zeolite membranes, SBF and 1.5SBF solutions were prepared according to the procedure reported by Kokubo and Takadama (2006). 150 mL of SBF was added to PET bottle and then the zeolite membrane was soaked in and collocated in an incubator at 37 °C for 21 days. Two routes of immersion were evaluated: (1) the solution of SBF was refreshed every 7 days; and (2) after 7 days of immersion the SBF solution was replaced by 1.5 SBF solution, which was refreshed every 7 days. The pH was monitored during the immersion period. For comparative purposes, the obtained zeolitic crystals together with zeolite membrane were pressed and soaked in SBF for 21 days.

### Characterization techniques

The morphology of the zeolite seeds and membranes was analyzed by SEM using a Jeol microscope (model JSV-6610LV). The crystalline phases were determined by XRD using a Bruker Diffractometer D8 Advance. The pH was measured using an Orion pH meter STAR 211. The FTIR analyses were performed using a Perkin Elmer Frontier spectrometer with Universal ATR sampling accessory which allows the analysis of solid and liquid samples. The FTIR spectra were recorded in the range of 4000–400 cm^−1^, composed of eight scans.

## Results and discussion

### Zeolite seeds and substrate

**Fig. 1:**
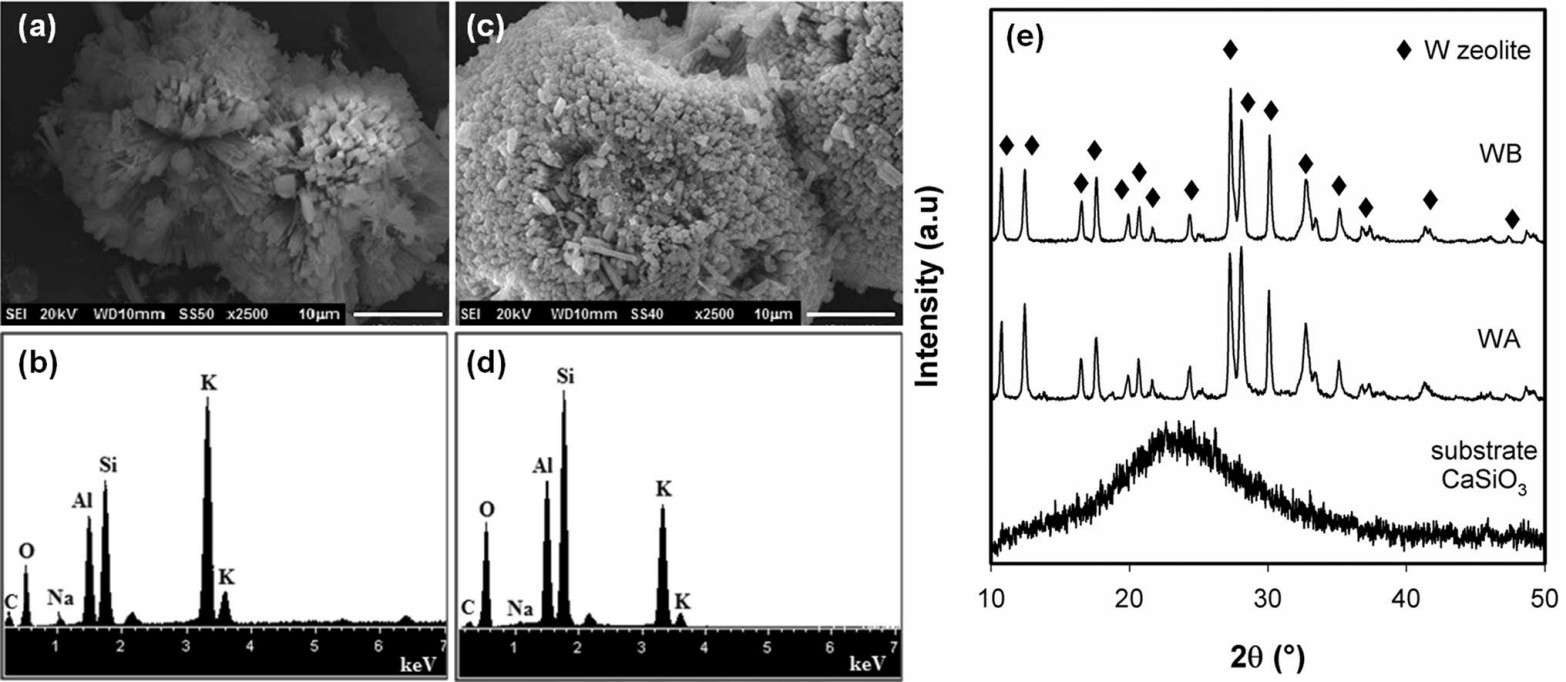
Micrographs and EDS spectra of (**a**, **b**) WA zeolite seed, (**c**, **d**) WB zeolite seed, and (**e**) XRD patterns of WA, WB zeolites and the substrate

The morphology, EDS spectra and XRD patterns of the WA and WB zeolite seeds and the support are shown in Fig. 1. The zeolite seeds exhibit a morphology of twin balls constituted by elongated crystals (Fig. 1a, c). The effect of quantity of sodium aluminate was observed in the morphology and size crystals. In case of higher content of aluminum, the twin balls were larger and the crystals were thinner compared to those observed in the zeolite seed synthesized with low content of aluminum (WA). The variation in Si/Al ratio influences the crystallization process modifying the nucleation and crystallization kinetics, the crystal size and morphology and the nature of the crystallization (Byrappa and Yoshimura 2001). The Si/Al ratio was of 1.44 and 1.83 for WA and WB zeolite seeds, respectively. With respect to XRD analysis (Fig. 1e), it can be observed that the calcium silicate substrate is an amorphous phase while the zeolite seeds were obtained as unique crystalline phase corresponding to Lynde W zeolite (JCPDS-86-1110).

### Zeolite membranes

**Fig. 2:**
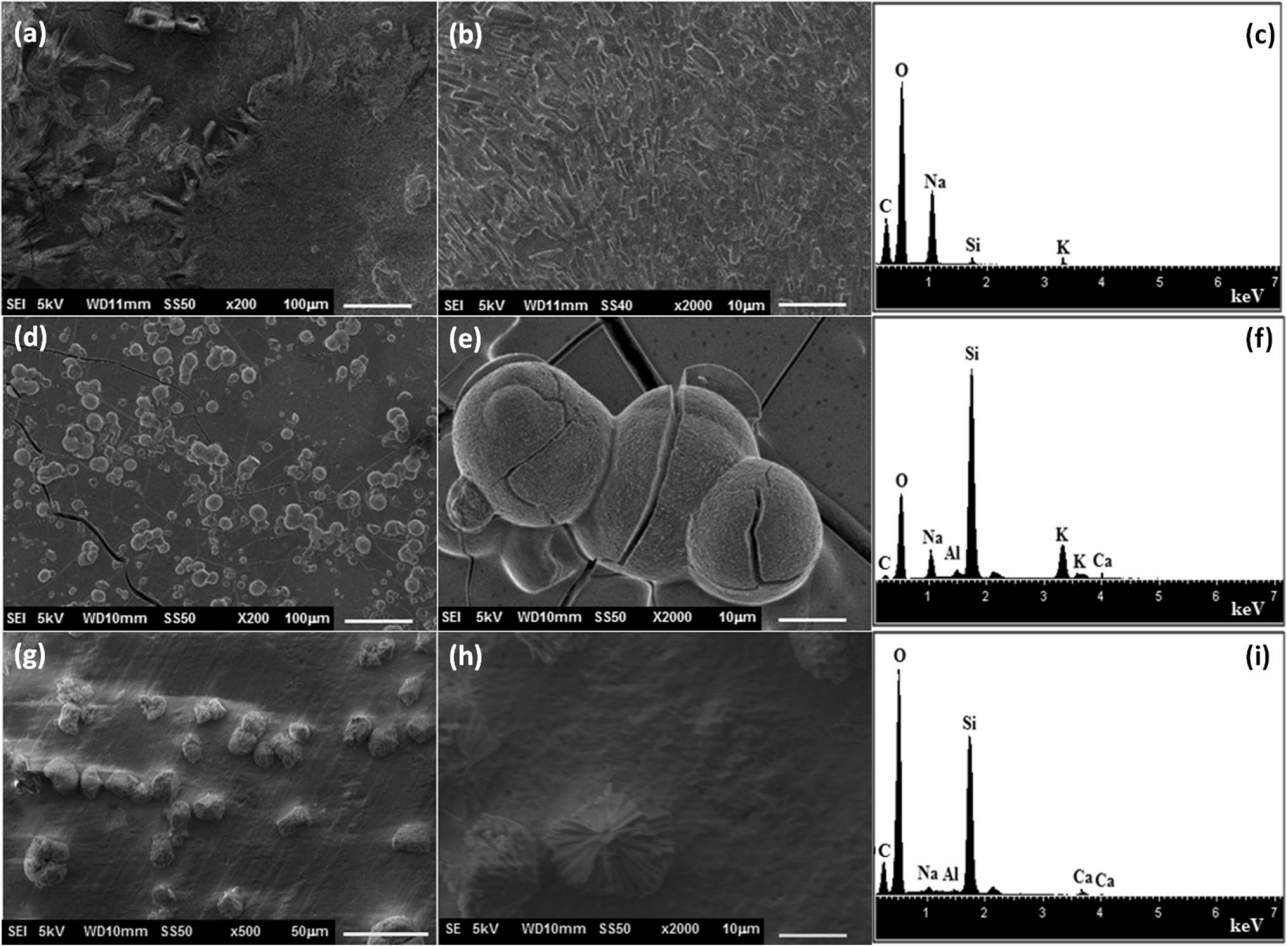
Micrographs and EDS spectra of zeolite membrane obtained on calcium silicate functionalized with (**a**–**c**) APS, (**d**–**f**) PEI and (**g**–**i**) PDDA after hydrothermal treatment with batch composition of WA zeolite

According to SEM analysis (Fig. 2), the functionalized substrates submitted to hydrothermal treatment with WA composition batch did not lead to the formation of a zeolite membrane. The substrate functionalized by APS (Fig. 2a, b) was partially coated by a layer of rectangular bars, which according to the EDS analysis was mainly constituted by sodium and oxygen. In case of the substrate functionalized with PEI (Fig. 2d, e), a vitrified layer was formed onto surface substrate and some twin-ball crystals were observed. The EDS analysis indicated that this vitrified layer is mainly constituted by silicon and oxygen. In case of the substrate functionalized with PDDA, only a few twin-ball crystals were observed, the elements of Ca, O and Si were detected by EDS, which correspond to the chemical composition of the substrate.

**Fig. 3:**
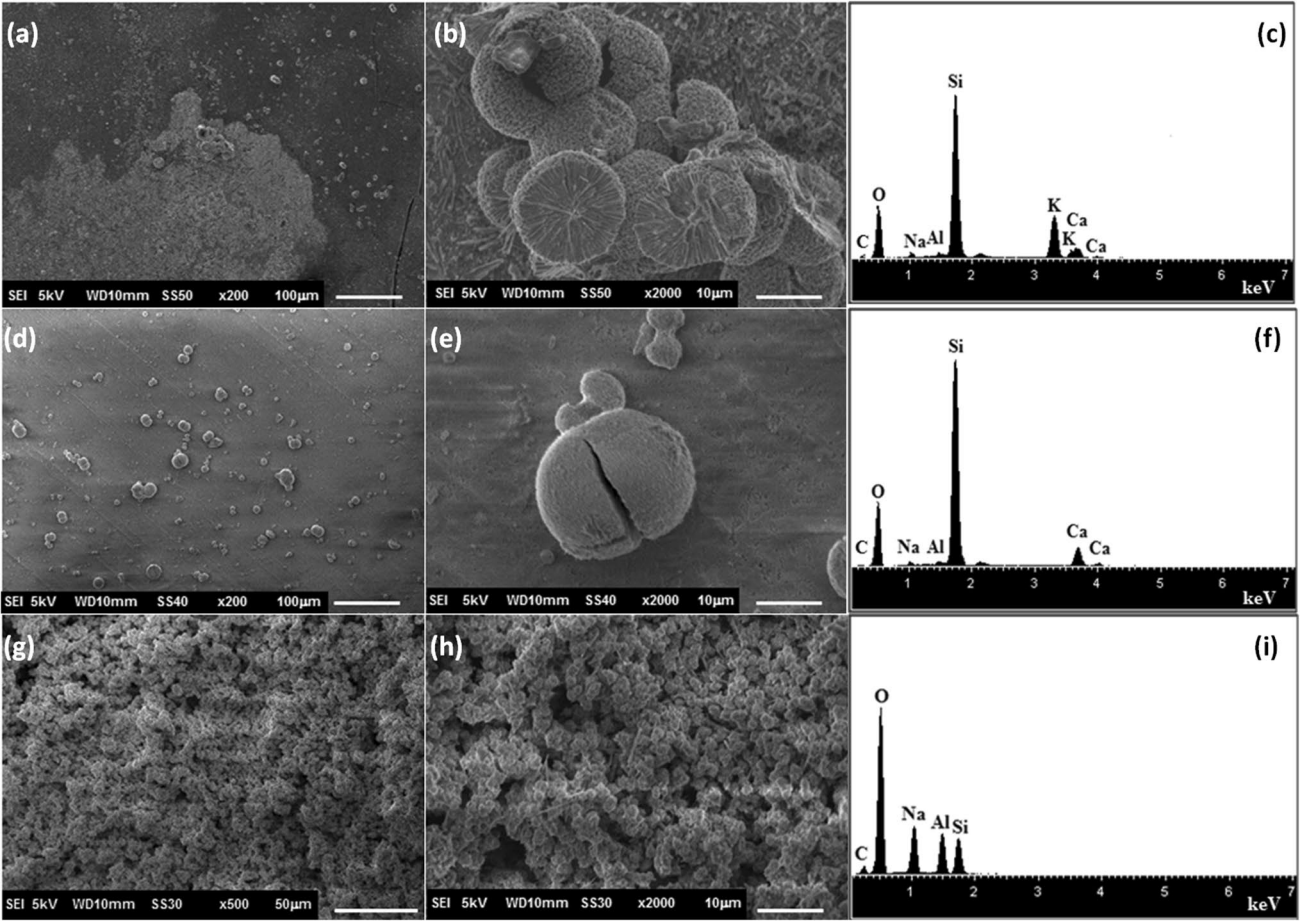
Micrographs and EDS spectra of zeolite membrane obtained on calcium silicate functionalized with (**a**–**c**) APS, (**d**–**f**) PEI and (**g**–**i**) PDDA after hydrothermal treatment with batch composition of WB zeolite

In Fig. 3, the micrographs of the functionalized substrates submitted to hydrothermal treatment with WB batch composition are shown. It can be observed that few twin-ball crystals were dispersed on the surface substrates functionalized with PEI and APS. By EDS analysis, the characteristic elements of the substrate (Si, O, Ca) were detected. On the other hand, the surface of PDDA-functionalized substrate (Fig. 3g) was covered by homogeneous layer of crystals which was constituted by silicon, aluminum, sodium and oxygen, elemental characteristic of the zeolitic phase. These crystals exhibited spherical and needles-like morphologies.

**Fig. 4:**
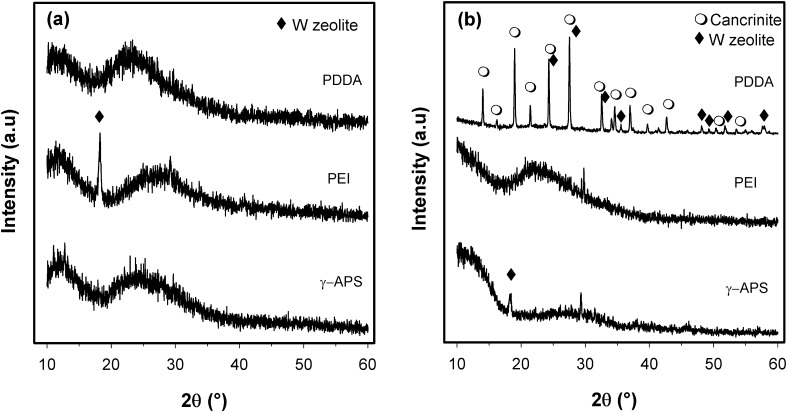
XRD patterns of zeolite membrane obtained on calcium silicate functionalized with different polyelectrolytes after hydrothermal treatment with batch composition of **a** WA zeolite and **b** WB zeolite

The zeolite membranes were characterized by XRD in Fig. 4 which shows the XRD patterns for both batch compositions (WA and WB). In case of WA batch composition (Fig. 4a), the membranes exhibit an amorphous phase corresponding to calcium silicate substrate and only a peak is observed in the PEI-functionalized substrate which is associated with W zeolite. Similar patterns have been obtained for PEI- and APS-functionalized substrate with the WB batch composition. In case of PDDA-functionalized substrate, two zeolitic phases are being identified: cancrinite (JCPDS 01-073-6315) corresponding to zeolite of formula Na7.14 Al_6_ Si_7.08_ O_26.73_ (H_2_O)_4.87_ and W zeolite. The formation of cancrinite (CAN) together with the W zeolite can be related to the effect of Si/Al ratio of batch composition, the nature of cationic polymer (PDDA) and the chemical nature of the substrate. Particularly, the calcium silicate substrate can interact with the batch composition and it can modify the Si/Al ratio of the reaction media enhancing the formation of a specific zeolite. According to the literature (Lopez-Orozco et al. 2011), the substrate can be reactive during in situ crystallization which may provide builders for zeolite framework. Alkali reaction media have led to the partial dissolution of silicate generating a different Si/Al ratio and releasing calcium which could contribute to the crystallization of a new zeolitic phase, the cancrinite. It is reported (Byrappa and Yoshimura 2001) that the composition and concentration of the reaction media, temperature, pressure and surface contact of the phases are some of the parameters which determine the rate of dissolution of the nutrient, mass transport and the possibility of new phases formation.

The differences obtained in the zeolite membrane formation applied in cationic polymer can be related to their functional groups and the way they interact with the support. In basic theories, it is assumed that the surface of silicate phases is negatively charged (Zingg et al. 2008). The inversions charge in the Stern layer can be induced depending on the chemical environmental of the solutions (Viallis-Terrise et al. 2001). According to the most reports (Singh and Ojha 1981; Chen and Mehta 1982), no charge inversion is observed for silicate phases in very dilute systems and negative zeta potentials have been measured in systems with low ionic strength at pH > 11. These findings agree with the pH changes observed during the functionalization of the support. The PDDA solution has presented an initial pH of 3.53 while APS and PEI have exhibited a basic pH 10.86 and 11.00, respectively. When the substrate was added to polymer cationic solution the pH was increased. In case of PDDA, the pH was 9 whereas in case of PEI the pH was 11.55, and in case of APS no significant changes were observed. This behavior could explain the homogeneous growth of the zeolite membrane on the support functionalized with PDDA, where cationic polymer enhances the inversion charge of the calcium silicate. Particularly, the PDDA generates a positive charge on the surface support during hydrothermal treatment promoting the adhesion of zeolite nuclei precursors leading to homogenous zeolite layer formation (Aguado et al. 2011).

On the other hand, the effect of Si/Al ratio of W zeolites observed on the formation of membrane is related to the surface properties of these materials. The zeta potential of zeolites depends on the pH, the ionic strength of the media and the aluminum content (Kuzniatsova et al. 2007). Additionally, the Si/Al ratio is related to electronegativity of the zeolite surface; the higher content of aluminum atoms there would be greater electronegativity of the zeolite (Jha and Singh 2016). This behavior was observed in this study for the W zeolite with low Si/Al ratio (1.44) which did not lead to formation of the zeolite membrane, while WB zeolite (Si/Al = 1.83) enhanced the growing homogeneous zeolite membrane. Thus, it is possible to infer that although the PDDA functionalization contributes to inverse the surface charge of the support, this modification is not enough to attract the precursor nuclei of the W zeolite of low Si/Al ratio. However, additional studies should be performed to clarify the role of these parameters on the formation of zeolite membrane.

**Fig. 5:**
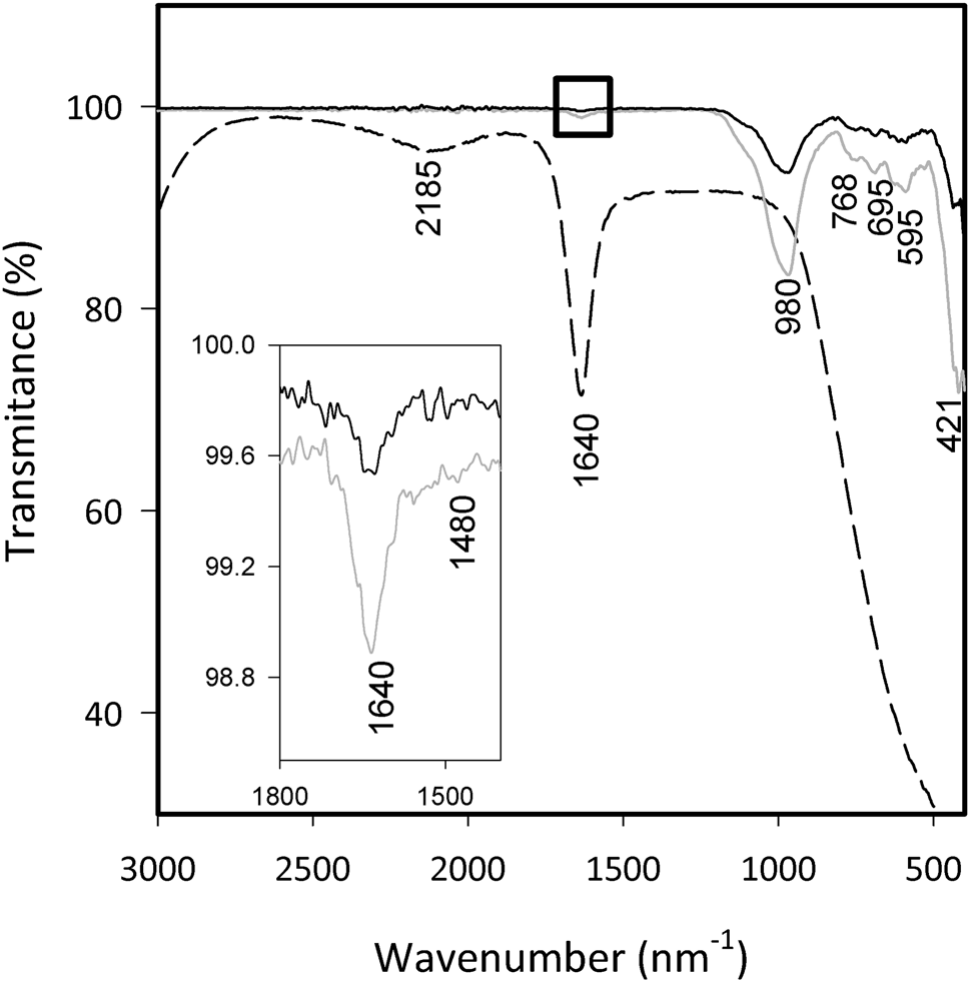
FTIR spectra of WB seed (black line), CAN–W membrane (gray line) and PDDA (medium dash line)

To identify whether functional groups from PDDA were incorporated in the CAN–W membrane, an analysis was carried out by FTIR to compare PDDA and the WB seeds. It can be observed in Fig. 5 that the WB seeds and CAN–W membrane present the bands associated with internal tetrahedral (1250–920 cm^−1^), T–O bend external linkages 650–500 cm^−1^ and pore opening vibrations in the region of 1150–1050 cm^−1^ (Liu 2009). The PDDA (medium dash line) has exhibited the characteristic bands of CH_*n*_ (2923–2925 cm^−1^) (Fu et al. 2015; Liu et al. 2010). Specifically, the band at 1640 cm^−1^ associated with C=C vibration is present in the CAN–W membrane as well as in the PDDA, which is of a higher intensity compared to WB seeds (without functionalization). Additionally in the inset, a band at 1480 cm^−1^ corresponding to vibrations of CH_2_ (Fu et al. 2015) is observed which indicates that the calcium silicate surface has been successfully functionalized with PDDA enhancing the growing of the zeolite membrane.

### Bioactivity evaluation of the zeolite membranes

**Fig. 6:**
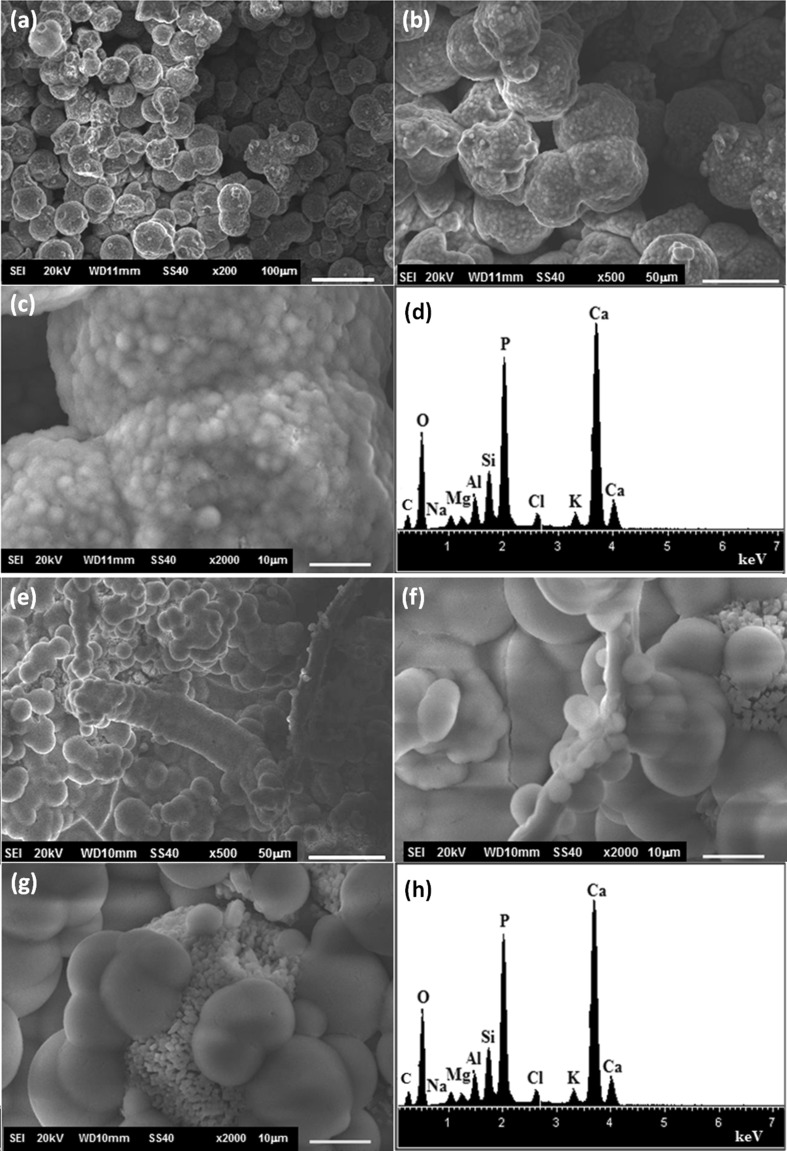
Micrographs and EDS spectra of W–CAN membrane after its immersion in **a**–**d** SBF for 21 days and **e**–**h** SBF (7 days) + 1.5SBF (14 days)

The bioactivity of CAN–W membrane was evaluated in SBF for 21 days. After immersion period, it was observed that the zeolite membrane was covered by a layer which was mainly constituted by calcium and phosphorous (Fig. 6). An effect of the immersion process was observed by zeolite membrane soaked in SBF for 21 days (Fig. 6a–c), during which the growth of Ca–P layer was evident in each single crystal of the zeolite, while in the immersion for 7 days in SBF and 14 days in 1.5 SBF (Fig. 6e–g) the ceramic layer was growing to cover the whole zeolite membrane. The morphology was dependent on the immersion process, and by re-immersion a spherical apatite layer was formed, while the simple immersion led to the formation of sponge type. These results indicate that the immersion method modifies the kinetics of nucleation of the ceramic layer, which is related to concentration, saturation, and pH. Additionally, the zeolite membrane is found stable during the immersion period that makes it useful for specific applications in the biomedical area. An accurate analysis by EDS has shown Ca/P ratio of 1.16 and 1.35 for SBF and re-immersion methods, respectively.

**Fig. 7:**
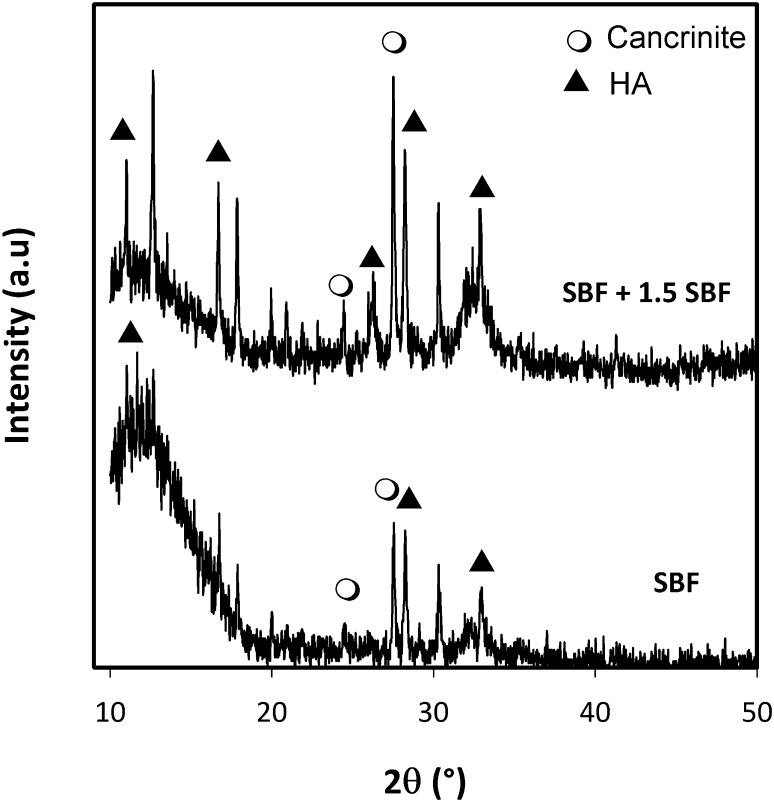
XRD patterns of CAN–W membrane after immersion in SBF for 21 days

**Fig. 8:**
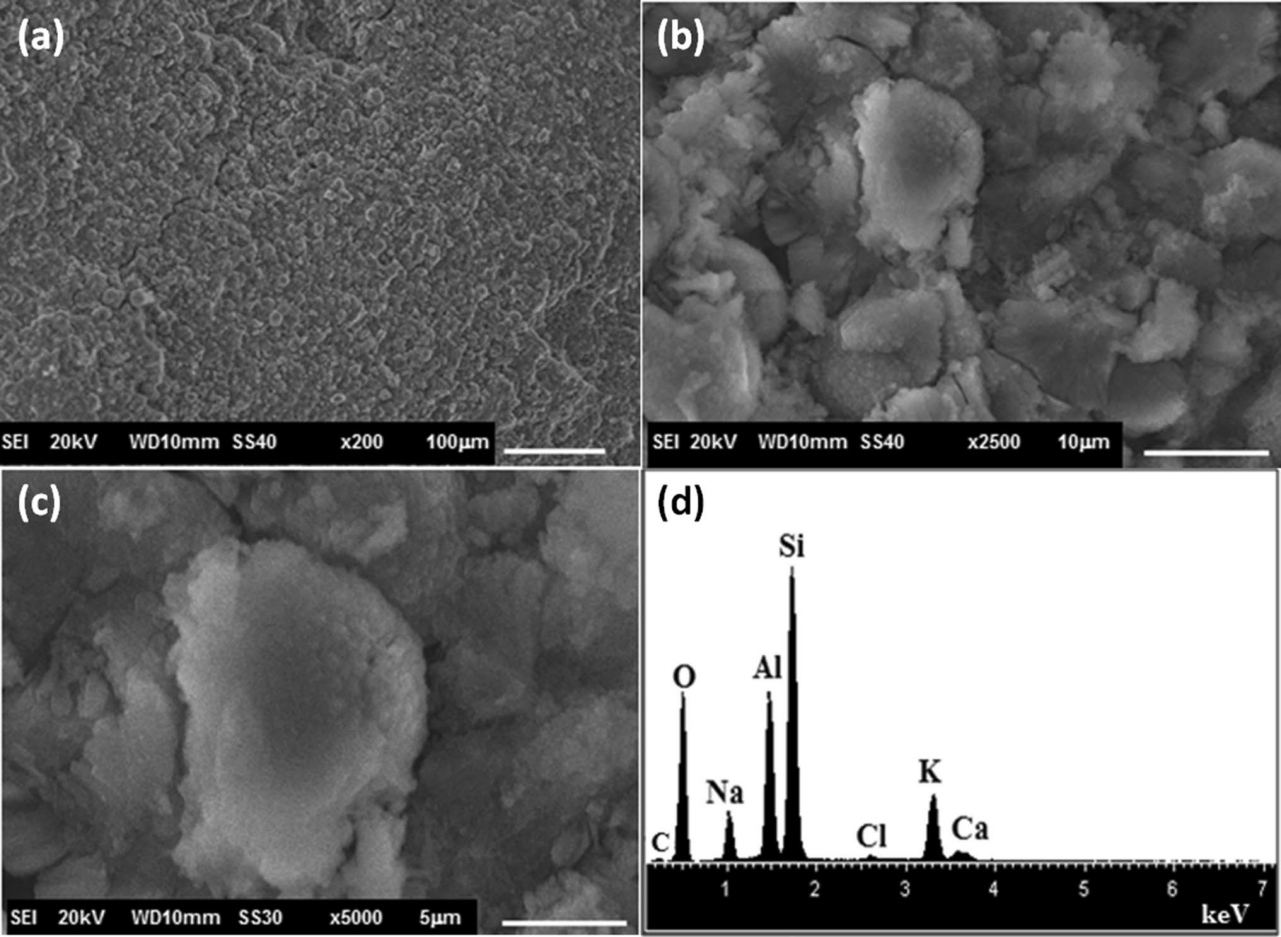
**a**–**c** SEM micrographs at different magnifications and **d** EDS spectrum of the W–CAN crystals after their immersion in SBF for 21 days

According to XRD analyses (Fig. 7), the ceramic layer has been formed on zeolite membrane corresponding to apatite phase, an indication of the bioactive response of the membrane. Additionally, peaks corresponding to cancrinite and W zeolite phases have been detected; it allows to infer that the thickness of the coating Ca–P is thin. The intensity of apatite peaks was higher for zeolite membrane soaked in by re-immersion method indicating that the ceramic coating was thicker than those soaked in SBF only. Besides, the re-immersion process seems to have accelerated the growth of the nuclei formed during the first 7 days in SBF. Figure 8 shows the SEM micrograph of the CAN–W zeolite crystals after its immersion in SBF. It can be observed that there is no apatite layer formation on the zeolite surface. By EDS analysis, the main peaks correspond to the elemental characteristic of the zeolite material; only as peak of low intensity attributed to calcium by indicating that CAN–W zeolite is not bioactive by itself. These results indicate that the synergic effect of substrate-zeolite enhances the formation of apatite layer. This approach allows a combination of properties of calcium silicate and zeolite phase, making this membrane a potential material for drug delivery and bone regeneration.

## Conclusions

A homogeneous zeolite membrane on calcium silicate substrate was obtained by a second growth method. The effect of the chemical nature of polyelectrolyte and Si/Al ratio was decisive for the affinity between the zeolite nuclei precursors and the support. The interaction of the support and the functional groups of the PDDA promoted the attraction of precursor nuclei of the W zeolite of high Si/Al ratio, which were observed by FTIR. After immersion in SBF, the zeolite membrane was stable and led to the formation of Ca–P layer on its surface. The re-immersion method led to the formation of richer Ca/P layer (1.36).

The synergic effect of the support and zeolite membrane led to the formation of Ca–P-rich layer without requiring an ionic exchange treatment of the zeolite. The bioactive response of the CAN–W membrane makes this material potentially useful for bone regeneration and vector drug delivering.

Additional studies will be performed to evaluate the cytotoxicity and cell viability of the zeolite membrane to extend the applications of these materials.
